# Osteoid Osteoma of the Toe: A Rare Presentation with Diagnostic Challenges

**DOI:** 10.5334/jbsr.3250

**Published:** 2023-08-29

**Authors:** Angel Castillo-Fortuño, Ana Belen Larque, Daniel Poggio

**Affiliations:** 1 Department of Radiology, Hospital Clinic de Barcelona, Barcelona, Spain; 2 Department of Pathology, Hospital Clinic de Barcelona, Barcelona, Spain; 3 Department of Orthopaedic Surgery, Hospital Clinic de Barcelona, Barcelona, Spain

**Keywords:** Osteoid osteoma, bone tumor, MRI, foot, toe

## Abstract

**Teaching Point:** Osteoid osteoma is one of the most frequent benign bone tumors; however, when found in the toes it usually presents atypical clinical and radiological features including soft tissue swelling that can lead to misdiagnosis.

## Case History

A 20-year-old male presented with complaints of progressing swelling and mild disturbance on the tip of the second left toe, without a preceding history of trauma. No involvement of other joints or systemic manifestations were reported. A foot magnetic resonance imaging (MRI) was requested under the suspicion of soft tissue abnormality.

**Figure 1:**
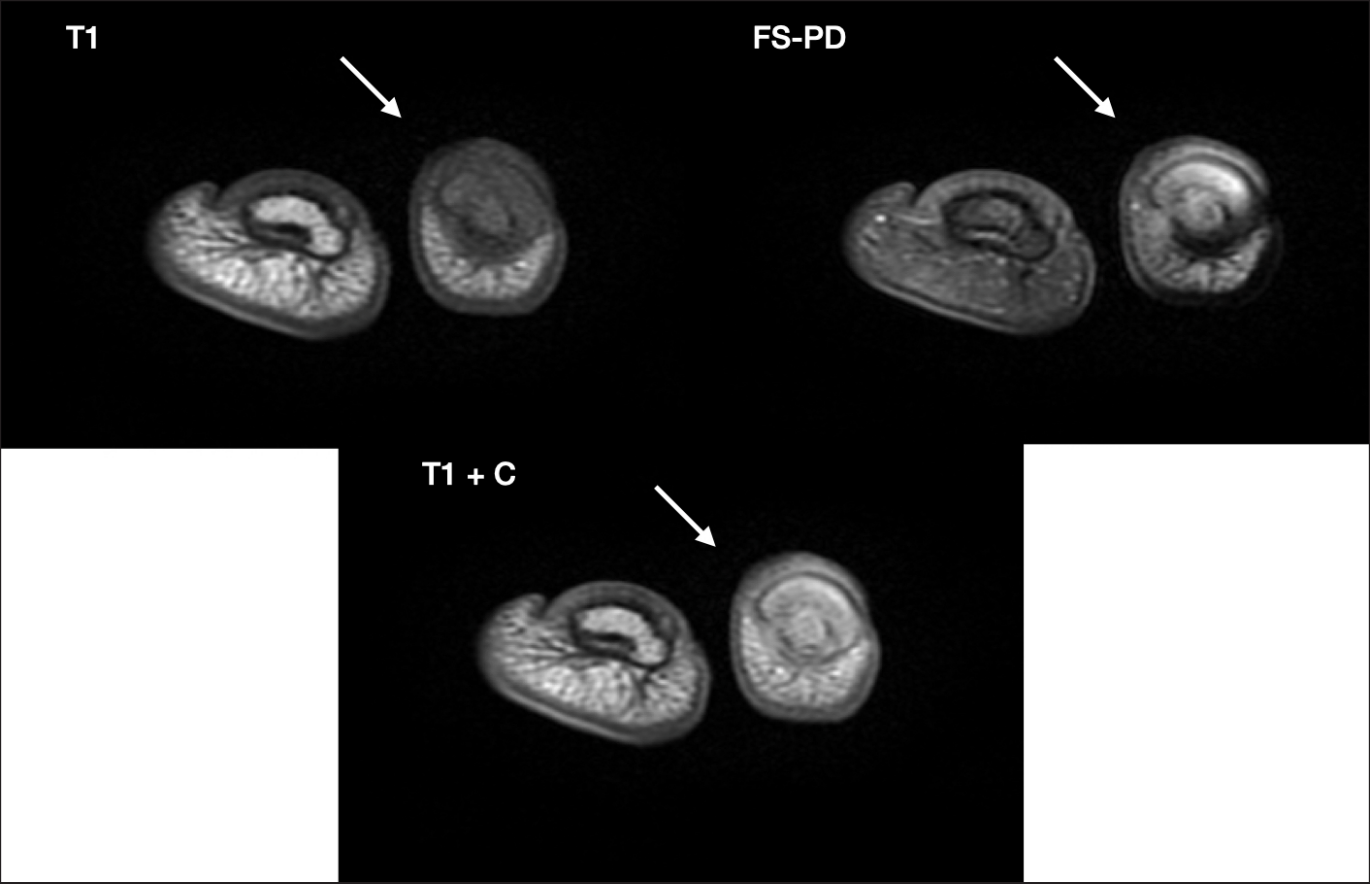


Magnetic resonance imaging showed a pseudonodular soft tissue subungual lesion with high signal on FS-PD images and contrast uptake (Figure 1).

**Figure 2:**
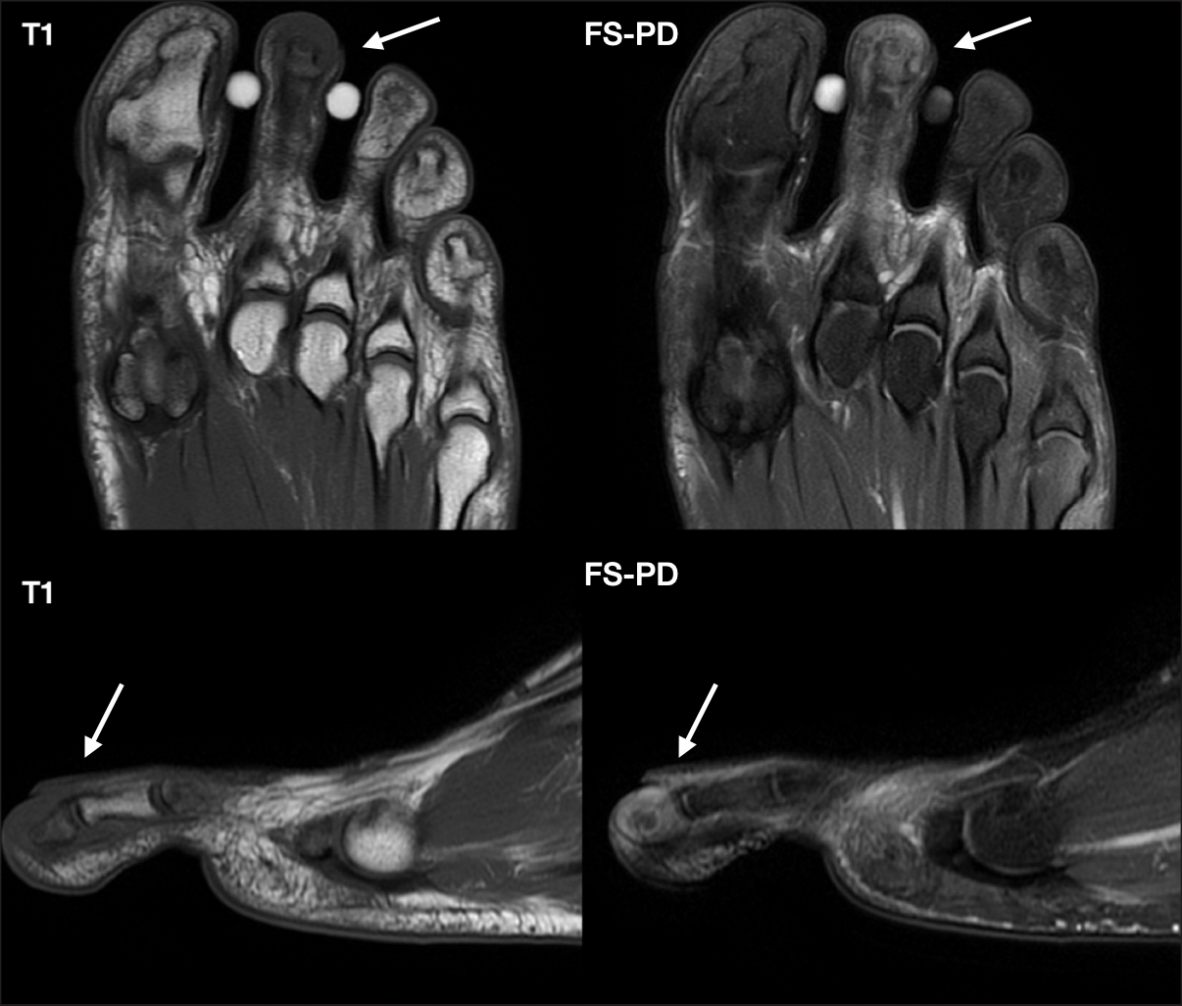


Associated findings included erosive changes on the terminal tuft and surrounding soft tissue edema (Figure 2). A presumptive diagnosis of glomus tumor was made.

Dermatologists performed surgical exploration of the subungual tissue, but no tumoral lesion was identified. Soft tissue samples were obtained without relevant findings.

Given the absence of a definitive diagnosis and the progressive development of morphological changes in the toe, it was decided to carry on with an excisional biopsy of the distal phalanx.

**Figure 3:**
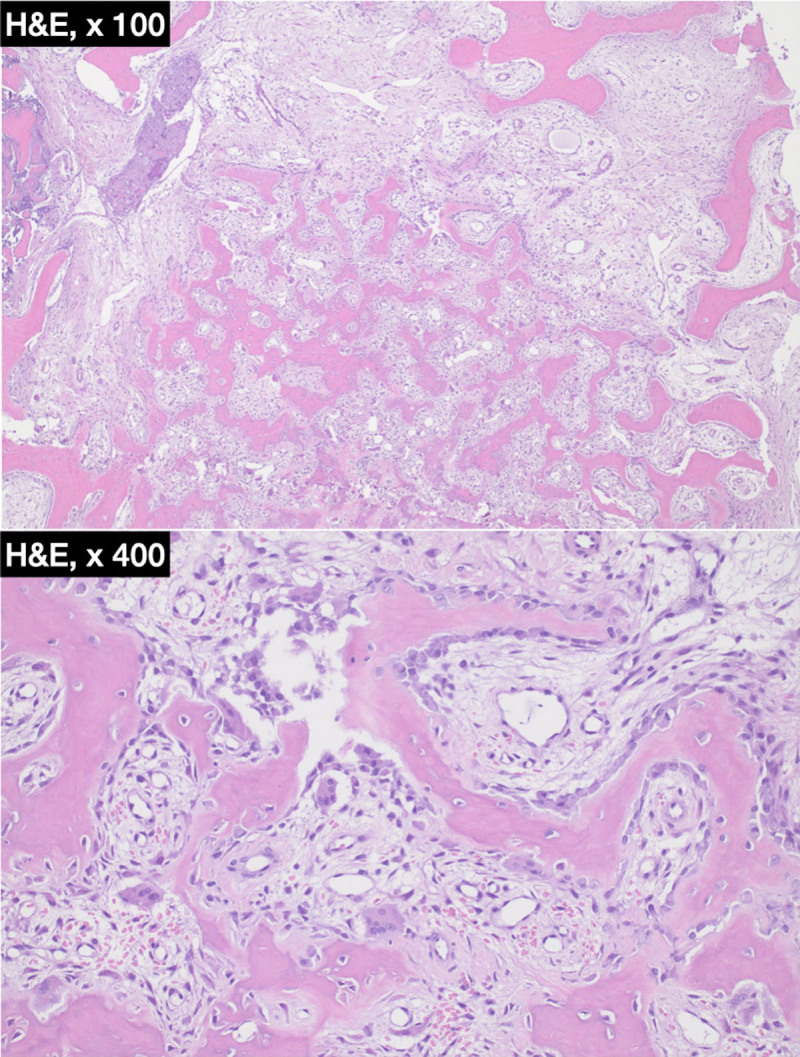


The patient was treated surgically, and histopathology study of the specimen showed anastomosing trabeculae of woven bone rimmed by plumb osteoblasts consistent with osteoid osteoma (Figure 3).

## Comments

Osteoid osteoma (OO) constitutes 10–12% of all benign bone tumors. It has a male predilection and usually occurs in young subjects. Patients typically present with a distinctive nocturnal pain relieved by non-steroidal anti-inflammatory medication. Radiologically, a particular feature is the presence of a cortical lucent nidus surrounded by sclerotic bony reaction [1].

OO of the toe has a low prevalence, being more frequent on the hallux and distal phalanges. Furthermore, when this lesion is found in the phalanges it is more likely to have an endosteal or periosteal location, which determines the uncommon clinical and radiological findings.

MRI studies of similar cases from literature only reported a characteristic nidus in half of them, probably because in small lesions the nidus and surrounding cortex show an indistinguishable signal intensity.

Less frequent pain of the lesion can be explained by the periosteal origin and the absence of weight-bearing stress. In addition, cutaneous and vasomotor alterations are directly related to the increased release of prostaglandins from the nidus.

All these clinical and radiological aspects derived from the OO in the toe phalanx must be differentiated from a wide range of conditions including infective, rheumatic, traumatic, and neoplastic circumstances [1]. Glomus tumor was the main suspected diagnosis in our patient because of the predominant subungual abnormalities and subtle bone findings.

The main treatment of OO is the removal of the nidus, either with surgical resection or by percutaneous procedures such as radiofrequency ablation.
